# A Joint Encryption and Compression Algorithm for Multiband Remote Sensing Image Transmission

**DOI:** 10.3390/s23177600

**Published:** 2023-09-01

**Authors:** Weijia Cao, Xiaoran Leng, Tao Yu, Xingfa Gu, Qiyue Liu

**Affiliations:** 1 Aerospace Information Research Institute, Chinese Academy of Sciences, Beijing 100094, China; lengxiaoran2022@163.com (X.L.);; 2 School of Remote Sensing and Information Engineering, North China Institute of Aerospace Engineering, Langfang 065000, China; liuqy_bhht@nciae.edu.cn

**Keywords:** remote sensing image transmission, image encryption, JPEG, 2D infinite collapse map, encryption-then-compression system

## Abstract

Due to the increasing capabilities of cybercriminals and the vast quantity of sensitive data, it is necessary to protect remote sensing images during data transmission with “Belt and Road” countries. Joint image compression and encryption techniques exhibit reliability and cost-effectiveness for data transmission. However, the existing methods for multiband remote sensing images have limitations, such as extensive preprocessing times, incompatibility with multiple bands, and insufficient security. To address the aforementioned issues, we propose a joint encryption and compression algorithm (JECA) for multiband remote sensing images, including a preprocessing encryption stage, crypto-compression stage, and decoding stage. In the first stage, multiple bands from an input image can be spliced together in order from left to right to generate a grayscale image, which is then scrambled at the block level by a chaotic system. In the second stage, we encrypt the DC coefficient and AC coefficient. In the final stage, we first decrypt the DC coefficient and AC coefficient, and then restore the out-of-order block through the chaotic system to get the correct grayscale image. Finally, we postprocess the grayscale image and reconstruct it into a remote sensing image. The experimental results show that JECA can reduce the preprocessing time of the sender by 50% compared to existing joint encryption and compression methods. It is also compatible with multiband remote sensing images. Furthermore, JECA improves security while maintaining the same compression ratio as existing methods, especially in terms of visual security and key sensitivity.

## 1. Introduction

“Belt and Road” is an abbreviation of the “Silk Road Economic Belt” and the “21st Century Maritime Silk Road”. Most “Belt and Road Initiative” countries are developing countries. Although they are faced with the need to develop natural resources and deal with natural disasters in the process of construction, it is difficult for them to use their independent space observation ability to meet these needs. Therefore, it is urgent to carry out sustainable development and application research of remote sensing technology by sharing remote sensing image data. To achieve this goal, it is necessary to transmit remote sensing image data. However, remote sensing image data are facing security risks in the transmission process. Remote sensing images carry extensive sensitive information about remote sensing targets [1]. Direct transmission of sensitive information is risky because hackers are usually interested in intercepting them [2,3]. Moreover, remote sensing image data transmission in countries along the “Belt and Road” involves cross-border/international transmission. These data often need to be transmitted to different countries and regions, which brings difficulties in control and protection [4].

Image encryption is an important method to ensure the security of image transmission. Currently, a wide range of image encryption methods have been developed and are available. These methods use various encryption technologies to ensure security and privacy, such as multimedia scrambling [5], wave perturbation [6], reversible cellular automata [7], bitplane-based image encryption [8], and chaos-based image encryption algorithms [9,10,11,12]. These image encryption algorithms can transform the original image into an incomprehensible distortion form, ensuring the security of the image during storage and transmission. However, with the continuous growth of the size of image files, the method of directly encrypting images will consume extensive computing resources. This problem will become more obvious when encrypting remote sensing images because of their larger amount of data. Since the countries along the “Belt and Road” are developing countries with limited computing resources, it is necessary to design a joint encryption and compression scheme to reduce the computational burden of these countries.

To encrypt and compress the transmitted images, the traditional joint encryption and compression scheme adopts the compression-then-encryption (CtE) system. This system compresses and encrypts data at the sender level first, and then decrypts and decompresses the data after transmission to the receiver. However, Zhou et al. pointed out that the CtE system gives all the tasks of encryption and compression to the sender; thus, it requires high computing resources of the sender, and the practical application scenarios are limited [13]. According to Zhou et al., in many scenarios, the primary concern of senders is typically the security of the image, while the efficient utilization of bandwidth may not be a significant consideration. Channel providers, on the other hand, are more concerned about how to reduce the amount of data transmitted and are less concerned about security. Therefore, they proposed an encryption-then-compression (EtC) system, which entrusted the compression task to the channel provider, saving the computing resources of the sender.

Although many existing EtC systems help the sender to save the cost of compression tasks, the sender still undertakes all encryption tasks, which will still bring problems in practical applications. In this case, all keys are generated by the sender; therefore, once the key is leaked at the sender level, the data will be at risk of being completely decrypted by the attacker. Moreover, most countries along the “Belt and Road” are developing countries with very limited computing resources. As the sender, it may be difficult for them to complete all encryption tasks.

**Figure 1:**
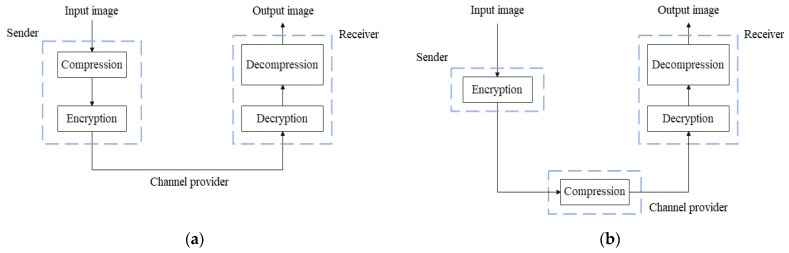

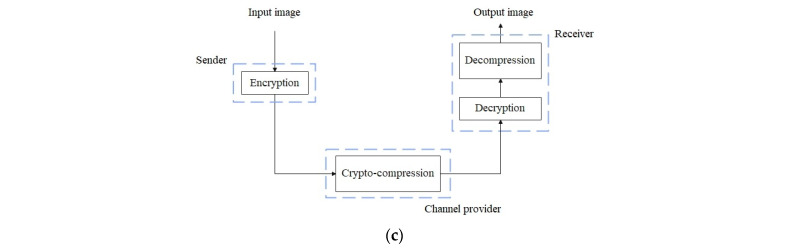
Data transmission scheme: (**a**) compression-then-encryption system; (**b**) encryption-then-compression system; (**c**) encryption-then-crypto-compression system.

To make up for the above shortcomings, we propose an encryption-then-crypto-compression system, as shown in Figure 1. This system needs the sender to undertake some encryption tasks that can meet basic security requirements, and the channel provider is responsible for further encryption and compression tasks. Using this scheme can not only reduce the risk of key leakage but also further save the computing resources of the sender.

In a previous study, we designed a chaotic mapping algorithm called a 2D infinite collapse map (2D-ICM) [14]. We proved that it has better chaotic performance than the existing chaotic maps through various tests. In addition, we also used 2D-ICM to scramble and confuse traditional digital images and achieved gratifying results in security. Therefore, in this paper, on the basis of the encryption-then-crypto-compression system, we design a joint encryption and compression algorithm (JECA) suitable for multiband remote sensing image transmission. In the preprocessing stage, we use 2D-ICM to scramble at the block level to realize the initial image encryption. In the stage of crypto-compression, we adopt a mixed encryption and compression method based on JPEG to realize further encryption and compression.

The main contributions of our article are as follows.

- An encryption-then-crypto-compression system is proposed, which is more suitable for remote sensing image data transmission with the “Belt and Road Initiative” countries. This system can meet the security and efficiency requirements of remote sensing image data transmission and save their computing resources as much as possible, which is achieved by handing over the encryption tasks to these countries and channel providers. In contrast, according to the existing EtC systems, the encryption tasks should be completed independently by these countries.
- Building on the encryption-then-crypto-compression system, a joint encryption and compression algorithm (JECA) is proposed. JECA is based on JPEG, solves the limitation that JPEG only processes three-channel digital images, and provides a solution for encryption and compression of multiband remote sensing images. By overcoming this challenge, JECA retains the excellent compression performance of JPEG and enables the encryption and compression of multiband remote sensing images.
- Compared with the existing EtC system, JECA further saves about 50% of the computing time of the sender and achieves a good balance between compression performance and data security.

## 2. Related Work 

### 2.1. JPEG

JPEG (Joint Photographic Experts Group) [15] is the most common digital image compression method, which is the most commonly used format for storing and transmitting photo images on the Internet [16]. We chose JPEG as the basis of JECA because it has excellent compression performance and a wide range of application scenarios. To better understand our scheme, it is necessary to introduce some key parts of the JPEG process.

For an input image, JPEG is mainly compressed through five steps: preprocessing, discrete cosine transform, quantization, zigzag scanning, and entropy encoding.

#### 2.1.1. Preprocessing

In the preprocessing step, the input image is converted from the RGB color space to the YCbCr color space. Y is the brightness part of the image, while Cb and Cr are the offsets of the blue and red chromaticity of the image, respectively. Then, to ensure the quality of the compressed image and the speed of data processing, it is necessary to divide the image after color space transformation into 8 × 8 blocks. Subsequent compression processes are carried out in blocks.

#### 2.1.2. Discrete Cosine Transform, Quantization, and Zigzag Scanning

Discrete cosine transform (DCT) uses a set of cosine functions with different frequencies and amplitudes to fit data. Its essence is the real part of the Fourier transform. It has the characteristics of energy concentration to concentrate important information together; thus, it is often used for image compression.

After the DCT of each block, the transform coefficients are concentrated in a small range in the upper left corner. The first coefficient in the upper left corner is called the DC coefficient, which has a large value and represents the low-frequency component in the block. The remaining 63 coefficients are called AC coefficients, which are small in amplitude and represent the high-frequency components of image information. The information of each block is concentrated in the low-frequency region; a higher-frequency region has a smaller coefficient. Therefore, the subsequent quantization operation can quantize these high-frequency components with small amplitude to 0, so as to exchange for higher compression performance at the expense of losing some accuracy.

**Figure 2:**
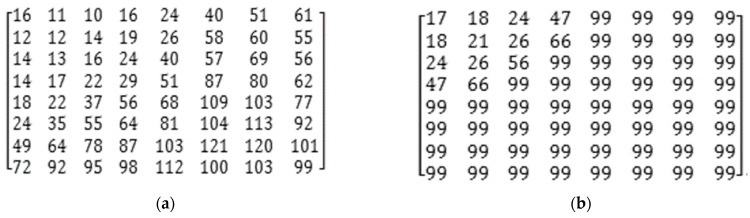
Standard quantization table provided by JPEG: (**a**) standard luminance quantization table; (**b**) standard chrominance quantization table.

Figure 2 shows the standard luminance quantization table and the standard chrominance quantization table provided by JPEG.

**Figure 3:**
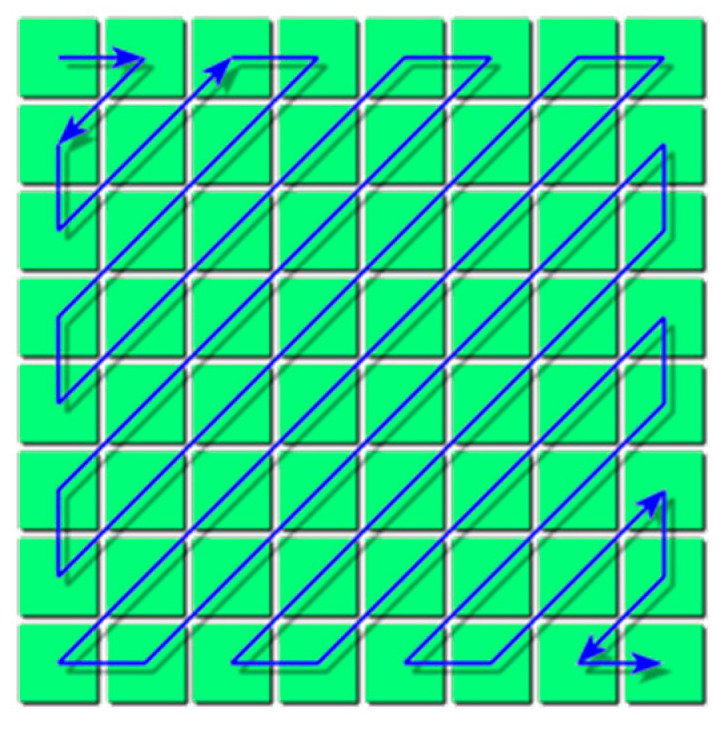
Zigzag scanning.

Since the quantized block is an 8 × 8 matrix, it should be converted into a one-dimensional vector by zigzag scanning, as shown in Figure 3. It can concentrate zero values as much as possible, which is convenient for subsequent entropy coding.

#### 2.1.3. Entropy Encoding

The last step of JPEG is to encode DC and AC coefficients into binary bitstreams. For quantized DC coefficients, the prediction error is calculated by taking the difference between the current DC coefficient and the DC coefficient of the previous adjacent block. For quantized AC coefficients, run-length encoding (RLE) is used to compress them. Then, the Huffman algorithm is used to encode the prediction error of the DC coefficient and the AC coefficient bitstreams after RLE. Finally, the compressed binary bitstreams are obtained.

### 2.2. Encryption and Compression Schemes Based on JPEG

Due to the excellent compression performance of JPEG, many schemes based on JPEG have been developed for the encryption and compression of digital images. According to the positioning of the encryption within the compression process, they can be classified into three categories: encryption before compression, joint compression and encryption, and compression before encryption [17]. Encryption before compression means that the image is encrypted before JPEG compression. Typical methods include permutation encryption [18] and some chaos-based encryption schemes that directly operate on the original image [19]. Kurihara et al. introduced a block scrambling-based encryption scheme [20]. Before compression, the input image is divided into blocks of equal size. Subsequently, various transformations are applied to these blocks, including scrambling, rotation, pixel flipping, and shuffling of color channels. Following the block-level encryption process, JPEG is applied to the transformed image. This method conforms to JPEG compression format and can ensure compression efficiency similar to JPEG. On this basis, Chuman et al. proposed an encryption scheme specifically designed for grayscale images [21]. In their approach, grayscale images are utilized as the input data for encryption. They used smaller block sizes and more block numbers in the encryption module, and then spliced the encryption results into grayscale images as the input of JPEG compression. This encryption scheme effectively reduces color information and gains stronger resistance to various attacks. The joint compression encryption algorithm combines compression and encryption techniques by incorporating encryption methods into one or more stages of the underlying compression process, such as encrypting DC and AC coefficients in the transformation stage [22,23,24,25,26,27], encrypting the quantization table in the quantization stage [28], and modifying bitstreams in the entropy coding stage [16,26]. Compression before encryption is to directly encrypt the JPEG compressed bitstreams. This method is usually compression-friendly; it will generate lower costs to send encryption keys [29,30,31]. However, Socek et al. pointed out that such systems are essentially non-format-compatible because the encryption methods of these systems usually directly process the compressed bitstreams, which is likely to destroy the compressed format [32].

Although the above schemes can ensure the good efficiency and security of data transmission, these methods cannot be directly applied to multiband remote sensing images because JPEG can only process three-channel digital images. To solve this problem, our algorithm preprocesses the input remote sensing images and merges them into gray images, effectively solving the limitation of JPEG regarding the number of image channels.

## 3. Proposed Method

The transmission scheme we designed mainly involves three objects: sender, channel provider, and receiver. The sender is mainly responsible for preprocessing and preliminary encryption of the input multiband remote sensing images. In our envisaged application scenario, the countries along the “Belt and Road” are senders. The channel provider is responsible for compressing and further encrypting the data, so as to improve the transmission efficiency and further ensure the security of the data during transmission. The receiver is responsible for decrypting and decoding the received data and reconstructing the multiband remote sensing image. From the transmission scheme we designed, it is not difficult to see that both the sender and the channel provider have to undertake some encryption tasks. In this way, not only can the risk of key leakage of the sender be reduced, but part of the calculation burden of the sender can also be reduced.

**Figure 4:**
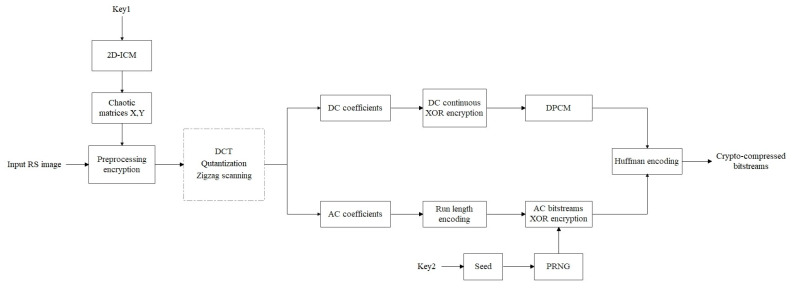
The overall structure of JECA.

Figure 4 illustrates the overall structure of JECA, which consists of three main modules: preprocessing encryption, DC continuous XOR encryption, and AC bitstream XOR encryption. The sender executes the preprocessing encryption module, and the channel provider executes other modules. As can be seen from Figure 4, the encryption task of JECA is mainly composed of preprocessing encryption of the sender, and DC coefficient and AC coefficient encryption of the channel provider. Adopting the way that the sender and the channel provider jointly undertake the encryption task can not only save the computing resources of the sender but also reduce the risk caused by the key leakage of the sender.

### 3.1. Preprocessing Encryption

In the preprocessing encryption stage, we combine all the bands of the input remote sensing image into a grayscale image and then use 2D-ICM to scramble this grayscale image based on blocks. JPEG can only support the input of three-band images at most, while the number of bands of remote sensing images is generally more than four. Therefore, we splice all bands of the input remote sensing image into a grayscale image, which effectively solves the limitation of JPEG on the number of input image channels. We choose 2D-ICM to perform block scrambling because it has excellent chaotic performance, which is very important to improve the security of encrypted images. Through these processes, we not only initially ensure the security of the input remote sensing image but also facilitate the subsequent crypto-compression process that the channel provider is responsible for.

**Figure 5:**
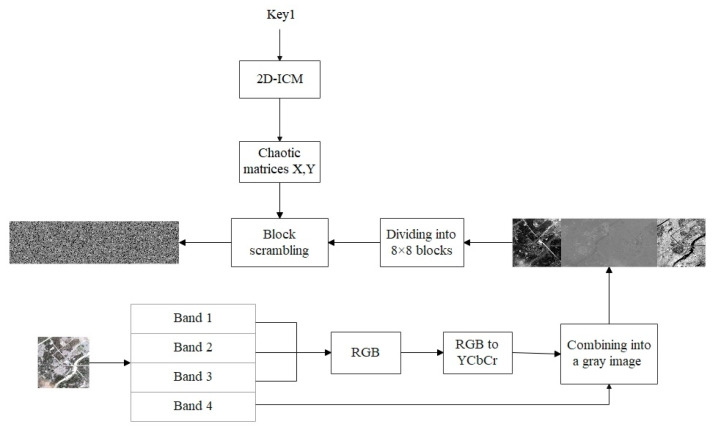
Preprocessing encryption of a four-band remote sensing image.

We take a four-band remote sensing image as an example of preprocessing encryption, which is shown in Figure 5. Assuming that the size of the input image is (x, y) and the number of bands is z, we put it into RGB channels in groups of three bands to transform the color space to YCbCr. Among them, if z is divisible by three, it means that all bands can be grouped; hence, all bands will undergo color space transformation. If z is not divisible by three, there will be remaining bands without grouping. The number of remaining bands can be recorded as m (0 ≤ m ≤ 2). These remaining bands will not be transformed into YCbCr.

After the previous processing, each group contains three single-band images, which correspond to Y, Cb, and Cr channels respectively, and the size of each single-band image is (x, y). We take the group as a unit, splicing three single-band images in each group into one grayscale image with the size of (x, 3y); if there are t groups, t grayscale images are obtained with the size of (x, 3y). Then, these t grayscale images are spliced together to obtain one grayscale image with the size of (x, 3yt). If there are m remaining bands, the grayscale image needs to be spliced together with the remaining bands, and we finally get a grayscale image with the size of (x, 3ytm). If there are no remaining bands, no further splicing is needed, and we finally get a grayscale image with the size of (x, 3yt).

Assuming that there are m remaining bands, the obtained grayscale image is divided into 8 × 8 blocks. The number of blocks N can be calculated using the following formula:

(1) $$N=\frac{x\times 3ytm}{64}.$$

Then, using the 2D-ICM [14] to generate chaotic matrices X and Y, the width and height of chaotic matrices can be obtained as follows:

(2) $$\left\{\begin{array}{c} \begin{array}{c} X_{w}=Y_{w}=\frac{x}{8}\;, \end{array}\\ X_{h}=Y_{h}=\frac{3ytm}{8}. \end{array}\right.$$

There is the following relationship between Equations (1) and (2):

(3) $$N=X_{w}\times X_{h}=Y_{w}\times Y_{h}.$$

**Figure 6:**
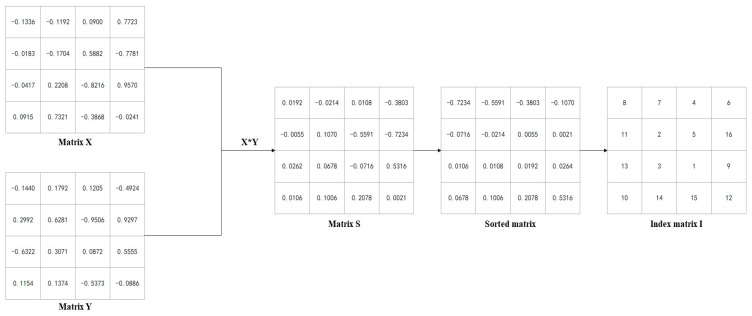
An example of generating index matrix I.

Finally, the generated chaotic matrices X and Y are used to scramble the distribution position of the blocks, which can be achieved by scrambling the index corresponding to each 8 × 8 block in the grayscale image. Figure 6 is an example of generating out-of-order index matrix I through chaotic matrices X and Y. Matrix S can be obtained by multiplying X and Y, and index matrix I can be obtained by arranging S in ascending order.

After block scrambling, the inter-block correlation of the image is greatly eliminated, which ensures the security of remote sensing image data before it is submitted to channel providers.

### 3.2. Crypto-Compression

After the sender submits the preprocessed remote sensing image data to the channel provider, the channel provider needs to compress and further encrypt it. They divide the obtained grayscale image into 8 × 8 blocks, with N blocks, and then perform discrete cosine transform, quantization, and zigzag scanning on each block. After performing the aforementioned operations, each block is converted into a one-dimensional array with a length of 64. The first value in the array represents the DC coefficient, which captures the overall intensity or brightness of the block. The remaining 63 values in the array represent the AC coefficients, which capture the variations or details within the block. Because the preprocessing encryption at the sender only eliminates the inter-block correlation of the image, the channel provider needs to further eliminate the intra-block correlation. This can be achieved by using different methods to encrypt DC and AC coefficients respectively.

#### 3.2.1. DC Continuous XOR Encryption

The encryption process of DC coefficients is carried out between blocks. Since each block contains one DC coefficient, the number of DC coefficients is N, which can be expressed as $\left\{{DC}_{1},{DC}_{2},{DC}_{3},\ldots ,{DC}_{N}\right\}$. We use the method of continuous XOR to encrypt these DC coefficients. The calculation formula is as follows:

(4) $${DC}_{i}={DC}_{1}⊕{DC}_{2}⊕{DC}_{3}\ldots ⊕{DC}_{i}\;,\;\;i=1,2,3,\ldots ,N.$$

This method changes all DC coefficient values except the first one; thus, it can effectively encrypt the brightness information of all blocks.

#### 3.2.2. AC Bitstream XOR Encryption

**Figure 7:**
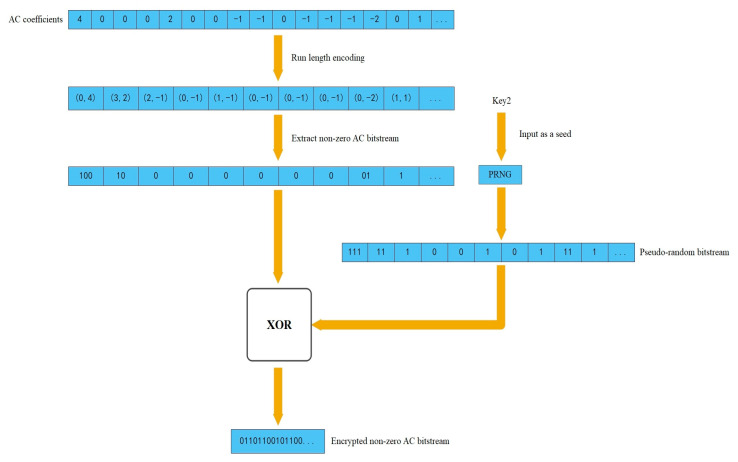
An example of the AC bitstream XOR encryption.

The encryption process of the AC coefficient is carried out inside the block. Each block contains 63 AC coefficients, which will be encoded with RLE. The RLE result can be expressed in the form of (a, b), where a is the number of consecutive zero values before the nonzero value, and b is the nonzero value. Figure 7 shows an example of AC bitstream XOR encryption. After RLE, continuous zero values are grouped together and nonzero values are encoded into a bitstream in the form of anti-code. We utilize the key as a seed to generate a pseudo-random bitstream using a pseudo-random number generator (PRNG). This generated bitstream is then XORed with the nonzero AC bitstream.

Note that, to prevent the compressed format from being destroyed, it is necessary to ensure that the bitstream before and after encryption has the same length. That is, the lengths of the nonzero AC bitstream, pseudo-random bitstream, and encrypted non-zero AC bitstream are all the same. By encrypting the AC coefficient, we successfully hide the detailed information in each block. 

Through the cooperation of preprocessing encryption, DC coefficient encryption, and AC coefficient encryption, the inter-block correlation and intra-block correlation of remote sensing image data are eliminated at the same time, which provides a good guarantee for the security of remote sensing image data transmission.

### 3.3. Decoding Process

**Figure 8:**
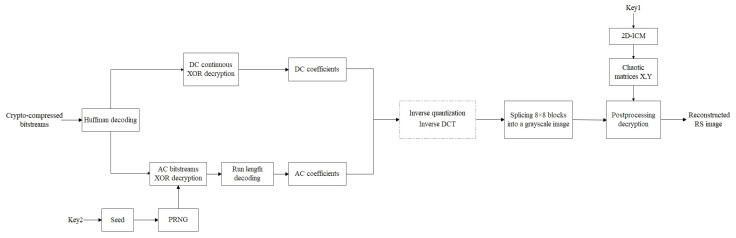
Decoding process.

The decoding process is the reverse of the compression and encryption process, aiming to recover the original image from the encrypted and compressed data. The decoding process is executed by the receiver. The overall structure of the decoding process is illustrated in Figure 8.

When the crypto-compressed bitstream is transmitted through the channel and received by the receiver, the decoding process is performed to recover the original image. The receiver decodes the crypto-compressed bitstream by the Huffman algorithm to obtain the encrypted DC coefficient and encrypted nonzero AC coefficient bitstreams. For the encrypted DC coefficient, the continuous XOR operation of Equation (4) is performed again to recover the DC coefficients. For the encrypted nonzero AC coefficient bitstream, a pseudo-random bitstream is first generated by Key2, and then a decrypted bitstream is obtained by the XOR operation similar to that in Figure 7. Finally, the decrypted bitstream is run-length decoded to obtain the reconstructed AC coefficients.

**Figure 9:**
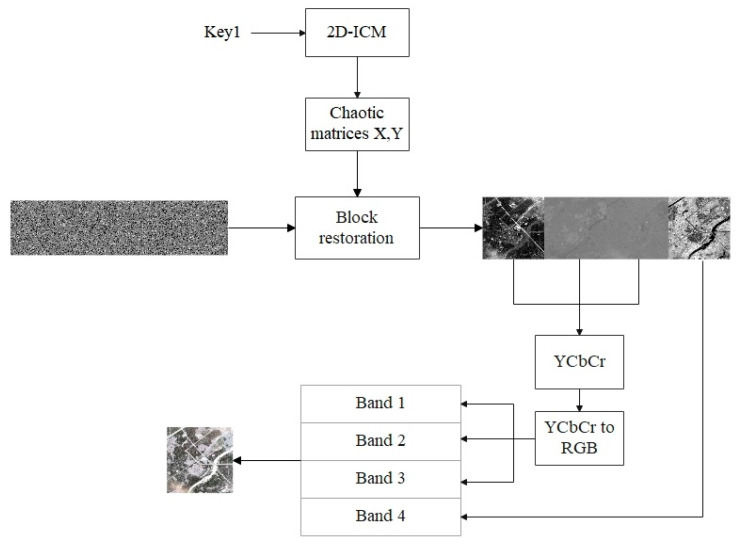
Postprocessing decryption of multiband remote sensing images.

After the DC coefficient and AC coefficient are obtained, they are reconstructed into matrices according to the order of zigzag scanning, and then the 8 × 8 blocks can be reconstructed by inverse quantization and inverse DCT. We again splice all the reconstructed blocks into a grayscale image. The remote sensing image can be reconstructed by postprocessing decryption, as shown in Figure 9.

Firstly, the chaotic matrices are generated, and the plaintext grayscale image can be restored using these two chaotic matrices to restore the arrangement order of blocks. Then, the restored grayscale image is split and put into YCbCr channels by the grouping method proposed in the preprocessing encryption stage. Finally, the data in the YCbCr channel are converted back into the RGB channel. This conversion process, combined with the grayscale image of the remaining bands, allows for the reconstruction of the complete remote sensing images.

## 4. Experimental Results

Qin et al. processed the remote sensing image data of 25 port cities along the “Belt and Road” taken by Tiangong-2 and made a multispectral image dataset [33]. The remote sensing images in the dataset are all 14 bands. We processed the dataset so that it could be used for subsequent comparative experiments.

First of all, we cropped all the remote sensing images, and only 1024 × 1024 pixels in the upper left corner were reserved in each band. Then, we quantized all remote sensing images into 8 bits. Assuming that the image before quantization is $S_{16}$, the formula of 8 bit quantization is as follows:

(5) $$S_{8}=\left\lfloor \;\frac{S_{16}-\min(S_{16})}{\max\left(S_{16}\right)-\min(S_{16})}\times 255\right\rfloor ,$$

where min ($S_{16}$) represents the minimum pixel value in the image, and max ($S_{16}$) represents the maximum pixel value in the image; $\left\lfloor \;\cdot \;\right\rfloor$ denotes rounding down.

After quantization, we took the 10th to 13th bands in the remote sensing image and finally made a dataset for the subsequent experimental test. We selected five test images with distinct characteristics for our experiments. These images were named Test 1, Test 2, Test 3, Test 4, and Test 5, respectively.

### 4.1. Security Test

In this section, we mainly test the security of JECA in terms of visual security, key sensitivity, and digital number distribution.

#### 4.1.1. Visual Security

**Figure 10:**
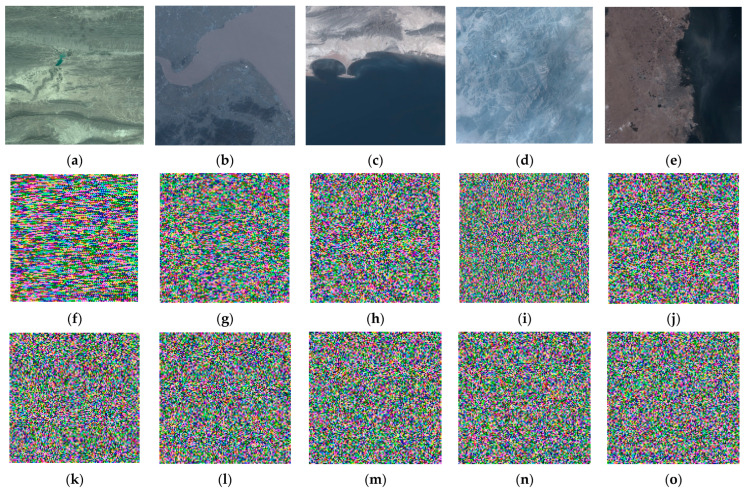
Visual effects of five remote sensing images before and after encryption: (**a**–**e**) images before encryption: (**f**–**j**) images not decrypted by the key of the sender: (**k**–**o**) images decrypted by the key of the sender.

In Figure 10, we test the visual effects of five encrypted images with and without the key of the sender to decrypt the encrypted images. It can be seen that, no matter whether the key has leaked or not, the attacker cannot intuitively infer any information from the encrypted image.

Moreover, we used the peak signal-to-noise ratio (PSNR) and structural similarity (SSIM) [34] to quantitatively describe the visual distortion of encrypted images. PSNR can be calculated by the following formula:

(6) $$PSNR=10\times {log}_{10}\left(\frac{255^{2}}{MSE}\right).$$

Generally speaking, a smaller PSNR value indicates that the encrypted image has a higher level of distortion compared to its original version. A higher visual distortion results in a smaller PSNR value and better visual security of the image. In addition, SSIM can also measure the visual distortion level of images. The range of SSIM is (0, 1]. A closer SSIM value to zero indicates higher visual distortion and better visual security of the image.

We calculated the PSNR and SSIM values of test images encrypted by JECA and other similar algorithms. The results are presented in Table 1 and Table 2.

Because JECA supports processing multiband remote sensing images, we also tested the visual effects when encrypting 14-band images. From the comparative data, we can see that, compared with other algorithms, JECA had the lowest PSNR and SSIM when encrypting three-band remote sensing images, and it could still maintain similar results when encrypting 14-band images. This means that our algorithm can have obvious visual distortion and high visual security whether when encrypting three-band remote sensing images or when encrypting multiband remote sensing images.

#### 4.1.2. Key Sensitivity

Key sensitivity means that, when two similar keys are used to encrypt one image, completely different encrypted images can be generated, and the encrypted images cannot be decrypted by similar but not identical keys. A secure encryption algorithm should exhibit key sensitivity, meaning that even a small change in the encryption key should result in a significant difference in the encrypted output.

To evaluate the key sensitivity of the encryption process, we encrypted Test 1 using two keys (key1 and key2). These keys had only 1 bit of difference. By comparing the generated encrypted images, we could assess the impact of key variations on the encryption process. Then, we used key1 and key2 to decrypt the same encrypted image to test the key sensitivity of the decryption process. 

**Figure 11:**
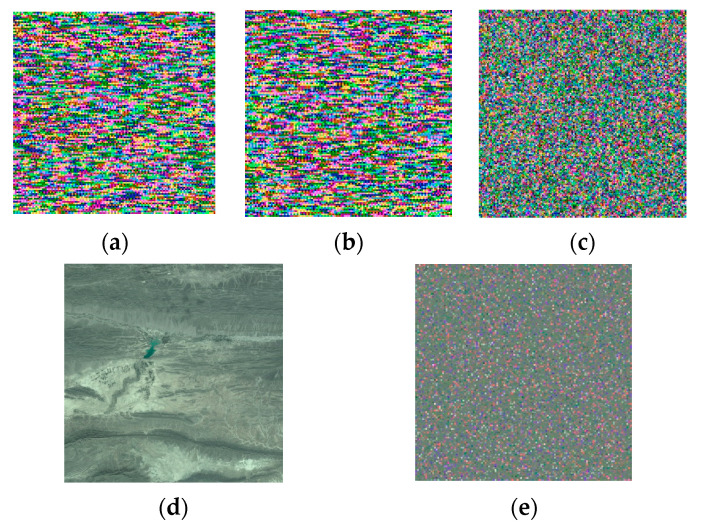
Key sensitivity test: (**a**,**b**) encrypted images generated by key1 and key2, respectively; (**c**) difference between (**a**,**b**); (**a**,**d**) decrypted with key1; (**a**,**e**) decrypted with key2.

The results are shown in Figure 11c as the result of the difference between Figure 11a,b. It can be seen that Figure 11a,b are completely different encrypted images, which proves the key sensitivity of the encryption process. Figure 11d,e are the results of decrypting Figure 11a with key1 and key2, respectively. It can be seen that the same key can be used to decrypt the image, but similar keys cannot be used for decryption, which proves the key sensitivity of the decryption process.

#### 4.1.3. Digital Number Distribution

**Figure 12:**
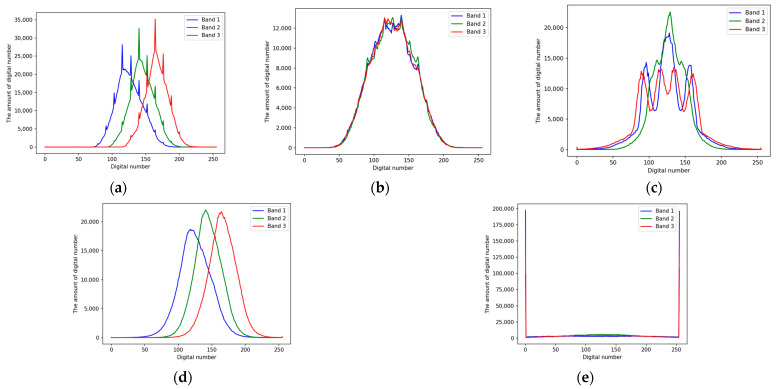
Digital number distribution curves of images encrypted by different algorithms: (**a**) digital number distribution of image Test 4; (**b**) digital number distribution of image encrypted by Kurihara’s method [20]; (**c**) digital number distribution of image encrypted by Chuman’s method [21]; (**d**) digital number distribution of image encrypted by Shimizu’s method [35]; (**e**) digital number distribution of image encrypted by JECA.

In statistical attacks, attackers will use a method based on digital number statistics to study the relationship between original images and encrypted images without knowing the key. Then, they will use the obtained relationship to predict images or to reduce the keyword search space for brute force attacks [36]. To resist statistical attacks, the digital number distribution of encrypted images must be different from that of original images. A greater difference in digital number distribution ensures a stronger ability to resist statistical attacks. To analyze the distribution characteristics of the encrypted images, we selected Test 4 as the test image and encrypted it using JECA and other algorithms, as shown in Figure 12.

The blue, green, and red curves represent the distribution of digital numbers in the first, second, and third bands, respectively. Notably, the digital number distribution of the image encrypted by our algorithm showed a significant deviation from that of the original image. This result demonstrates the strong resilience of our algorithm against statistical attacks, as it introduces a high level of distortion in the digital numbers.

### 4.2. Time Complexity

We tested the computing resource consumption of the sender by calculating the time required for the sender to execute the encryption algorithm.

The experiment used Python 3.6 and was run in PyCharm 2020.3.3. The processor was AMD Ryzen 7 4800 h with Radeon graphics 2.90 GHz, and the graphics card was NVIDIA GeForce GTX 1650Ti. We counted the time required for JECA to encode at the sender level, and compared it with typical EtC systems based on JPEG. 

The experimental results are shown in Table 3. Compared with the traditional EtC algorithm, JECA can spend a shorter encoding time at the sender. This means that JECA can greatly save the resource consumption of the sender.

### 4.3. Compression Performance

We used the CR-SSIM curve to test the compression performance of JECA and the quality of reconstructed images. A closer SSIM value to 1 indicates lower image distortion and a better quality of the reconstructed image. CR represents the compression ratio; the calculation formula is as follows:

(7) $$CR=\frac{the\;compressed\;image\;file\;size}{the\;original\;image\;file\;size}\times 100\%.$$

**Figure 13:**
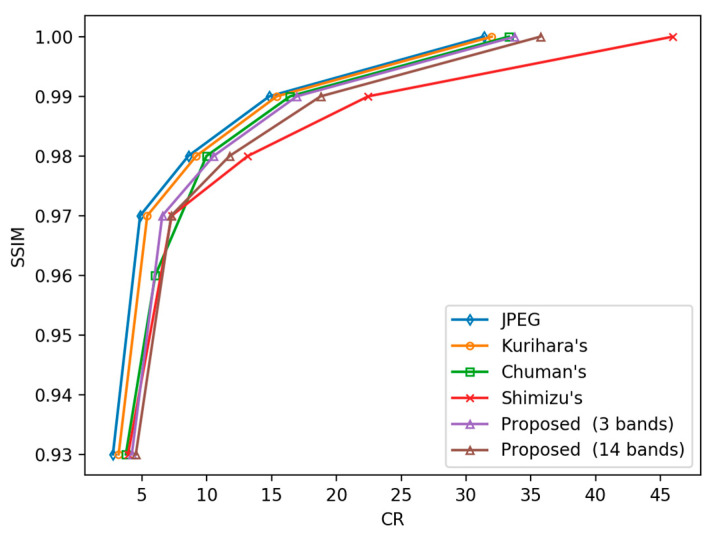
Performance comparison of algorithms JPEG, Kurihara’s [20], Chuman’s [21], Simizu’s [35] and our proposed.

The comparison curve of compression performance is shown in Figure 13. It can be seen that JECA could ensure compression efficiency similar to JPEG and other similar algorithms, whether when encrypting remote sensing images in three bands or when encrypting remote sensing images in 14 bands. Specifically, it could reduce the remote sensing image file size to about 5% of the original on the premise that the SSIM was close to 0.93.

## 5. Conclusions and Discussion

In this paper, we proposed a joint encryption and compression algorithm (JECA) for multiband remote sensing images, including a preprocessing encryption stage, crypto-compression stage, and decoding stage. In the first stage, the sender preprocesses and encrypts the input remote sensing image, and then obtains a grayscale image that disrupts the block arrangement order. In the second stage, the channel provider is responsible for compressing and further encrypting the grayscale image to obtain the encrypted binary bitstreams. In the decoding stage, the receiver is responsible for decoding and decrypting the received binary bit stream to obtain the reconstructed remote sensing image. The experimental results showed that JECA could reduce the preprocessing time of the sender by 50% than existing joint encryption and compression methods. Moreover, JECA could obtain higher visual security, key sensitivity, and resistance to statistical attacks on the premise of ensuring the compression performance similar to the existing joint encryption and compression methods.

At present, our proposed algorithm still has some limitations. First of all, because the framework we adopt is based on a lossy compression algorithm, we can only exchange a higher compression ratio at the expense of losing some image information. Although the missing information may not be visually obvious, it will produce additional errors in some experiments that require high-precision data. Consequently, we hope that, in the future, we can use a lossless compression algorithm to improve our scheme on the premise of ensuring high compression performance.

In addition, there are two problems when JPEG is used to process remote sensing images. One is that the number of channels cannot exceed three, and the other is that the bit depth cannot exceed 8 bits. In this paper, we provided a scheme that can solve the channel number limitation, but we did not solve the bit depth limitation, which is the reason why we needed to quantize the dataset with 8 bits. Therefore, in future work, we hope that our proposed scheme can process remote sensing images with higher bit depth.

## Figures and Tables

**Table 1:** PSNR comparison between JECA and other algorithms.

PSNR	Kurihara’s [20]	Chuman’s [21]	Shimizu’s [35]	JECA
Test 1	17.45	17.31	23.43	**8.74**
Test 2	15.14	14.92	29.54	**9.08**
Test 3	9.51	9.65	26.80	**7.22**
Test 4	15.40	15.55	24.53	**8.88**
Test 5	9.17	9.53	27.50	**7.12**
14-band images	-	-	-	**7.56**

**Table 2:** SSIM comparison between JECA and other algorithms.

SSIM	Kurihara’s [20]	Chuman’s [21]	Shimizu’s [35]	JECA
Test 1	0.29	0.25	0.31	**0.06**
Test 2	0.44	0.26	0.66	**0.09**
Test 3	0.29	0.13	0.71	**0.07**
Test 4	0.31	0.22	0.36	**0.07**
Test 5	0.28	0.14	0.64	**0.07**
14-band images	-	-	-	**0.05**

**Table 3:** Comparison of encoding time at the sender.

Time	Kurihara’s [20]	Chuman’s [21]	JECA
Test 1	0.668	0.635	**0.289**
Test 2	0.682	0.650	**0.281**
Test 3	0.686	0.634	**0.307**
Test 4	0.681	0.638	**0.290**
Test 5	0.698	0.640	**0.287**

All results are in seconds.

## Data Availability

The data presented in this study are available on request from the corresponding author.

## References

[1] Zhang X., Zhu G., Ma S. (2012). Remote-sensing image encryption in hybrid domains. Opt. Commun..

[2] Dong J., Wu G., Yang T., Li Y. (2018). The Improved Image Scrambling Algorithm for the Wireless Image Transmission Systems of UAVs. Sensors.

[3] Wang P., Wang Y., Xiang J., Xiao X. (2022). Fast Image Encryption Algorithm for Logistics-Sine-Cosine Mapping. Sensors.

[4] Lu S., Ye J., Tan Y. Research on the Security of Data Cross-border Circulation in Cyberspace. Proceedings of the 2023 International Conference on Distributed Computing and Electrical Circuits and Electronics (ICDCECE).

[5] Li C., Lin D., Feng B., Lu J., Hao F. (2018). Cryptanalysis of a Chaotic Image Encryption Algorithm Based on Information Entropy. IEEE Access.

[6] Wu Y., Zhou Y., Agaian S., Noonan J.P. (2014). A symmetric image cipher using wave perturbations. Signal Process..

[7] Wang X., Luan D. (2013). A novel image encryption algorithm using chaos and reversible cellular automata. Commun. Nonlinear Sci. Numer. Simul..

[8] Cao W., Zhou Y., Chen C.L.P., Xia L. (2017). Medical image encryption using edge maps. Signal Process..

[9] Zhu H., Zhao Y., Song Y. (2019). 2D Logistic-modulated-Sine-coupling-Logistic chaotic map For Image Encryption. IEEE Access.

[10] Zhu H., Qi W., Ge J., Liu Y. (2018). Analyzing Devaney Chaos of a Sine–Cosine Compound Function System. Int. J. Bifurc. Chaos.

[11] Zhu H., Zhang X., Yu H., Zhao C., Zhu Z. (2017). An image encryption algorithm based on compound homogeneous hyper-chaotic system. Nonlinear Dyn..

[12] Hanif M., Iqbal N., Ur Rahman F., Khan M.A., Ghazal T.M., Abbas S., Ahmad M., Al Hamadi H., Yeun C.Y. (2022). A Novel Grayscale Image Encryption Scheme Based on the Block-Level Swapping of Pixels and the Chaotic System. Sensors.

[13] Zhou J., Liu X., Au O.C. On the design of an efficient encryption-then-compression system. Proceedings of the 2013 IEEE International Conference on Acoustics, Speech and Signal Processing.

[14] Cao W., Mao Y., Zhou Y. (2020). Designing a 2D infinite collapse map for image encryption. Signal Process..

[15] Wallace G.K. (1991). The JPEG still picture compression standard. Commun. ACM.

[16] He J., Huang S., Tang S., Huang J. (2018). JPEG Image Encryption with Improved Format Compatibility and File Size Preservation. IEEE Trans. Multimedia.

[17] Li P., Lo K.T. (2020). Survey on JPEG compatible joint image compression and encryption algorithms. IET Signal Process..

[18] Gan Z., Chai X., Han D., Chen Y. (2018). A chaotic image encryption algorithm based on 3-D bit-plane permutation. Neural Comput. Appl..

[19] Chai X., Gan Z., Yuan K., Chen Y., Liu X. (2019). A novel image encryption scheme based on DNA sequence operations and chaotic systems. Neural Comput. Appl..

[20] Kurihara K., Shiota S., Kiya H. An encryption-then-compression system for JPEG standard. Proceedings of the 2015 Picture Coding Symposium (PCS).

[21] Chuman T., Sirichotedumrong W., Kiya H. (2018). Encryption-then-Compression Systems using Grayscale-based Image Encryption for JPEG Images. IEEE Trans. Inf. Forensics Secur..

[22] Korshunov P., Ebrahimi T. Scrambling-based tool for secure protection of JPEG images. Proceedings of the IEEE International Conference on Image Processing (ICIP).

[23] Li S., Zhang Y. (2016). Quantized DCT coefficient category address encryption for JPEG image. KSII Trans. Internet Inf. Syst. (TIIS).

[24] Puteaux P., Wang Z., Zhang X., Puech W. Hierarchical High Capacity Data Hiding in JPEG Crypto-compressed Images. Proceedings of the 2020 28th European Signal Processing Conference (EUSIPCO).

[25] Ong S., Wong K., Qi X., Tanaka K. (2015). Beyond format-compliant encryption for JPEG image. Signal Process. Image Commun..

[26] Qian Z., Zhou H., Zhang X., Zhang W. (2016). Separable Reversible Data Hiding in Encrypted JPEG Bitstreams. IEEE Trans. Dependable Secur. Comput..

[27] Liang H., Zhang X., Cheng H. (2019). Huffman-code based retrieval for encrypted JPEG images. J. Vis. Commun. Image Represent..

[28] He K., Bidan C., Le Guelvouit G., Feron C. (2016). Robust and secure image encryption schemes during JPEG compression process. Electron. Imaging.

[29] Cheng H., Zhang X., Yu J., Zhang Y. (2016). Encrypted JPEG image retrieval using block-wise feature comparison. J. Vis. Commun. Image Represent..

[30] Zhang D., Zhang F. (2014). Chaotic encryption and decryption of JPEG image. Opt. —Int. J. Light Electron Opt..

[31] Zhang X., Cheng H. Histogram-based retrieval for encrypted JPEG images. Proceedings of the 2014 IEEE China Summit & International Conference on Signal and Information Processing (ChinaSIP).

[32] Socek D., Kalva H., Magliveras S.S., Marques O., Culibrk D., Furht B. (2007). New approaches to encryption and steganography for digital videos. Multimed. Syst..

[33] Qin B., Li L., Xuan S., Li S., Liu K. (2020). Multi-spectral image data set of port cities along the “Belt and Road” taken by Tiangong-2. J. Remote Sens..

[34] Wang Z., Bovik A.C., Sheikh H.R., Simoncelli E.P. (2004). Image quality assessment: From error visibility to structural similarity. IEEE Trans. Image Process..

[35] Shimizu K., Wang Q., Suzuki T. AC Prediction Error Propagation-based Encryption for Texture Protection of JPEG Compressed Images. Proceedings of the 2021 Picture Coding Symposium (PCS).

[36] Taneja N., Raman B., Gupta I. (2012). Combinational domain encryption for still visual data. Multimedia Tools Appl..

